# Reducing anxiety and depression in Chinese adolescents through group behavioral activation: a pilot study with school-based implementation

**DOI:** 10.1186/s13034-025-01012-1

**Published:** 2025-12-24

**Authors:** Fang Zhang, Wenjing Liu, Hongmei Yang, Yang Sun, Xiaoxia Lei, Yue Ding, Xiaochen Zhang, Zhishan Hu, Shuaishuai Hu, Zhen Wang, Wenhong Cheng

**Affiliations:** 1 Department of Child and Adolescent Psychiatry, Shanghai Mental Health Centre, Shanghai Jiao Tong University School of Medicine, 600# South Wan Ping Rd., Xu Hui District, Shanghai, 200030 China; 2 School of Psychology, Shanghai Jiao Tong University, Shanghai, 200030 China; 3 Shanghai Jing’An Institute of Education, Shanghai, 200070 China; 4 Shanghai Key Laboratory of Emotions and Affective Disorders, Shanghai Jiao Tong University School of Medicine, Shanghai, 200025 China

**Keywords:** Group behavioral activation therapy, Adolescents, Anxiety, Depression, School-based intervention, Preventive mental health

## Abstract

**Background:**

Anxiety and depression are increasingly prevalent public health concerns among adolescents. Group Behavioral Activation Therapy (GBAT), a structured school-based intervention, has shown promise as a potential approach for alleviating these conditions.

**Methods:**

This quasi-experimental trial evaluated the efficacy of school-implemented GBAT in reducing anxiety and depressive symptoms among Chinese adolescents. Participants (*N* = 139; aged 12–17 years; 44.6% male) were assigned to either a GBAT group (*n* = 72) or a waitlist control group (*n* = 67). The GBAT protocol consisted of eight weekly 90-minute sessions. Outcomes were assessed at baseline, post-intervention, and 3-month follow-up using the Screen for Child Anxiety Related Emotional Disorders (SCARED) and the Depression Self-Rating Scale for Children (DSRSC).

**Results:**

A total of 93.06% of students completed all eight GBAT sessions. Post-intervention SCARED scores decreased significantly, *t* (135) = 2.44, *MD* = 4.20, *95% CI* [0.79, 7.60], *p* = 0.016, *Cohen’s d* = 0.42. Linear mixed-model analysis revealed a significant group effect (*F* (1,397) = 10.60, *p* = 0.001), indicating lower anxiety scores in the GBAT group compared with the waitlist control. A significant time effect was also observed, *F* (1,397) = 7.64, *p* < 0.001. At 3-month follow-up, GBAT maintained significant improvement in anxiety (SCARED: *MD* = 8.69, *95% CI* [3.58, 13.80], *p* < 0.001 *Cohen’s d* = 0.82) and depressive symptoms (DSRSC: *MD* = 2.28, *95% CI* [0.10, 4.46], *p* = 0.037, *Cohen’s d* = 0.43), indicating moderate to large effect sizes.

**Conclusions:**

These preliminary findings suggest that GBAT may be a promising primary prevention strategy for adolescent mental health. This study provides a foundation for future research exploring its potential integration into school-based mental health frameworks.

## Introduction

Anxiety and depression are common mental health disorders among adolescents, with prevalence rates rising markedly in recent years [1]. According to the World Health Organization [2], the global prevalence of anxiety disorders among adolescents is 4.1% for those aged 10–14 years and 5.3% for those aged 15–19 years, while the prevalence of depression is 1.3% and 3.4%, respectively. This concerning trend is mirrored, and potentially exacerbated, in low- and middle-income countries [3, 4].

Depression and anxiety substantially impair social, academic, physical, and family functioning [5, 6]. During adolescence, these conditions significantly increase the risk of subsequent mental health disorders, educational or employment difficulties, substance abuse, and suicidal behavior [7]. Importantly, even subthreshold anxiety and depression are linked to adverse outcomes, including increased disease burden, functional impairment, and elevated suicide risk [8–10]. Furthermore, delaying intervention for anxiety and depressive symptoms can accelerate disease progression and the development of comorbidities, considerably increasing the risk of poor prognosis [6, 11]. The substantial comorbidity and symptom overlap between anxiety and depression underscore the neurobiological and clinical rationale for transdiagnostic interventions that more efficiently target their shared mechanisms [12, 13].

Given their rising prevalence and significant impact, preventing and mitigating anxiety and depression among adolescents is an urgent public health priority. However, a large proportion of adolescents do not receive needed care due to multifaceted barriers, including stigma, poor symptom recognition [14], shortages in the mental health workforce [15], limited access to help-seeking information [16], and structural obstacles such as scheduling conflicts and financial constraints [17]. These systemic treatment-access barriers are also especially severe in low- and middle-income countries such as China [18, 19], highlighting the urgent need for scalable preventive interventions. Schools offer an efficient platform for early prevention and intervention, leveraging advantages such as eliminating transportation challenges, enabling access to large numbers of students, and improving time/cost-effectiveness [20–23]. Empirical evidence supports the feasibility and effectiveness of such school-based prevention programs [24]. Furthermore, group therapy yields outcomes comparable to individual therapy while allowing greater efficiency and broader reach, enhancing its feasibility and potential for effective implementation within school settings [25, 26]. This approach is particularly well-suited to schools in China, which face limited professional resources yet serve large student populations.

Behavioral Activation (BA) demonstrates significant implementation strengths due to its operational simplicity, explicit focus on behavioral change, and transdiagnostic applicability, particularly in targeting avoidance mechanisms [27]. Crucially, BA can be effectively delivered by non-specialists and demonstrates greater cost-effectiveness and cross-cultural adaptability than traditional cognitive behavioral therapy [28–31]. These features enhance its viability for low-resource settings. However, empirical evidence supporting its efficacy in adolescent populations, especially when delivered by non-specialists, remains limited.

GBAT, developed by Chu et al. [32], is a transdiagnostic cognitive-behavioral intervention featuring a structured, manualized group protocol for concurrently treating comorbid anxiety and depression. Designed with adaptability and accessibility as priorities, GBAT demonstrates strong implementation feasibility in educational settings [33]. Although originally validated in the United States [34], GBAT’s core components and delivery model align with resource-efficiency requirements in low- and middle-income countries. However, its clinical efficacy and implementation feasibility in these contexts remain to be empirically established. Therefore, this pilot study aims to evaluate the preliminary efficacy of GBAT, delivered by school mental health personnel, in reducing anxiety and depressive symptoms among Chinese adolescents.

## Methods

### Design

GBAT was implemented as an extracurricular mental health program across three pilot schools. Eligible middle school (grades 6–8) and senior secondary school students (grades 10–11) voluntarily enrolled after receiving study information. All participants completed standardized screening using the Screen for Child Anxiety Related Emotional Disorders (SCARED) [35, 36] and the Depression Self-Rating Scale for Children (DSRSC) [37, 38].

To accommodate substantial neurodevelopmental and institutional differences between educational stages, GBAT sessions were stratified by academic level (middle school vs. senior secondary). The intervention protocol strictly adhered to the standardized GBAT manual [33] and comprised eight 90-minute sessions with identical therapeutic content across cohorts. Core therapeutic components and session-specific implementation details are presented in Table 1. Symptom trajectories were evaluated at baseline, post-intervention, and the three months post-intervention using identical standardized instruments.

Students assigned to the control group were administratively deferred to a second-semester intervention cohort, establishing a natural waitlist control group during the active intervention phase. During this period, control participants engaged in standard school-based extracurricular activities (e.g., writing clubs, drama clubs) that were systematically screened to exclude therapeutic components or mental health-related content.

### Procedures

This quasi-experimental trial received ethical approval from the Institutional Review Board of the Shanghai Mental Health Center affiliated with Shanghai Jiao Tong University School of Medicine (Approval No. 2011-25). The ethical approval remained valid from 2011 to 2015 and renewed annually. All participants provided written informed consent in accordance with the Declaration of Helsinki. Baseline assessments were conducted in participating schools, where 1,617 students in grades 6–8 and 10–11 completed the SCARED and DSRSC. Within one week after assessment, 560 students who met provisional eligibility thresholds (SCARED ≥ 23 or DSRSC ≥ 15) were identified. School mental health personnel, in collaboration with homeroom teachers, applied predefined exclusion criteria: (1) active self-injurious behaviors, (2) need for immediate clinical intervention or frequent school absences (more than two days per week on average), or (3) apparent intellectual disability.

Eligible students and their parents/guardians received formal invitations detailing the GBAT program, explicitly stating that participation involved efficacy evaluations via standardized assessments. Final enrollment required (1) confirmed symptom thresholds (SCARED ≥ 23 or DSRSC ≥ 15); (2) written parental consent; and (3) student assent. Due to resource constraints, a clustered waitlist-controlled design was implemented. Eligible participants were first stratified by grade and then randomly assigned to receive GBAT either in Semester 1 or Semester 2 (waitlist control group). Randomization was performed using a simple procedure by an independent researcher not involved in participant recruitment, assessment, or intervention delivery.

All participants completed standardized psychometric assessments at three predetermined time points: (1) baseline (two weeks prior to intervention commencement); (2) post-intervention (within one week of program completion); and (3) three-month follow-up (within one week of the follow-up period). Assessments were implemented collaboratively by uniformly trained personnel, including master’s-level clinical psychology trainees and school mental health personnel, adhering strictly to identical protocols across all measurement occasions.

All GBAT facilitators held bachelor’s or master’s degrees in psychology, education, or related disciplines, and were certified as Level 2 Psychological Counselors, the minimum qualification for psychotherapy practice in China. Prior to delivering the intervention, facilitators completed an initial 32-hour workshop conducted by the GBAT development team. Subsequently, the training manual was culturally and linguistically adapted to the local context through a collaborative process involving the research team and all facilitators. A final 16-hour structured training session was conducted to review the adapted manual in detail, including demonstrations and supervised skill practice. Throughout the intervention period, all facilitators received weekly clinical supervision from licensed psychotherapists experienced with the protocol. To minimize the influence of pre-existing relational dynamics, power hierarchies, role expectations, and associated stereotypes, this study employed a cross-school delivery model. Under this design, school mental health personnel from one school delivered the intervention to student groups in schools other than their own. Furthermore, the traditional classroom seating was replaced with a round-table arrangement to foster an egalitarian and open group atmosphere.

**Table 1:** The content of GBAT

Sessions		Main content
Group session 1		Introduction; psychoeducation about anxiety, depression and other emotional problems
Group session 2		*See where I’m stuck*: self-assessment in functional domains; behavior and mood monitoring
Individual session 1		Enhancing motivation
Group session 3		*Keep active and keep approaching*: linking events with mood and anxiety; describe the “distress loop” and how avoidance perpetuates distress
Group session 4		*Identify meaningful goals*: goal setting *Look for ways to reach my goals*: individualized functional analysis
Individual session 2		*Identify meaningful goals*: goal setting (for students who have difficulty setting goals)
Group sessions 5–7		*Lasting change*: problem-solving; targeted behavioral activation and exposures
Group session 8		*See what’s worked*: reviewing progress and re-evaluate functioning across domains

### Outcomes

The primary outcome was the change in the total SCARED score after the 8-week intervention. Secondary outcomes included (1) response rates, defined as a ≥ 50% reduction in the total SCARED score from baseline, and (2) remission rates, defined as a total SCARED score ≤ 12 [39], along with changes in the DSRSC total score.

### Statistical analyses

Baseline demographic and clinical characteristics were compared using independent samples *t*-tests for continuous variables and *χ²* tests for categorical variables.

The primary outcome was analyzed using an independent samples *t*-test comparing pre- and post-intervention SCARED scores in the GBAT group. Secondary outcomes were compared between the GBAT and control groups using χ² tests.

Other outcomes were analyzed using linear mixed-effects models, adhering to intention-to-treat principles with all participants included. To enhance the robustness of these findings, we also conducted a supplementary per-protocol analysis for these continuous outcomes. The linear mixed-effects models were selected for their superiority over traditional repeated-measures ANOVA in handling missing data under the plausible Missing at Random assumption, thereby providing valid estimates without requiring data imputation related to attrition. The models specified fixed effects for group, time, and their interaction, with a random intercept for participants.

To address missing data comprehensively, a dual strategy was employed: (1) Item-level missingness on the SCARED and DSRSC scales was treated as Missing at Random and handled using Multiple Imputation by Chained Equations with 20 imputed datasets. The imputation model included only the items of the scales themselves and did not incorporate any covariates. The imputation was performed using the R package ‘mice’ with the default method (predictive mean matching), which is based on maximum likelihood principles, (2) attrition-related missingness was incorporated directly into the LMM framework. All other analyses were conducted using SPSS v.29 (IBM Corp.), with α = 0.05.

**Fig. 1:**
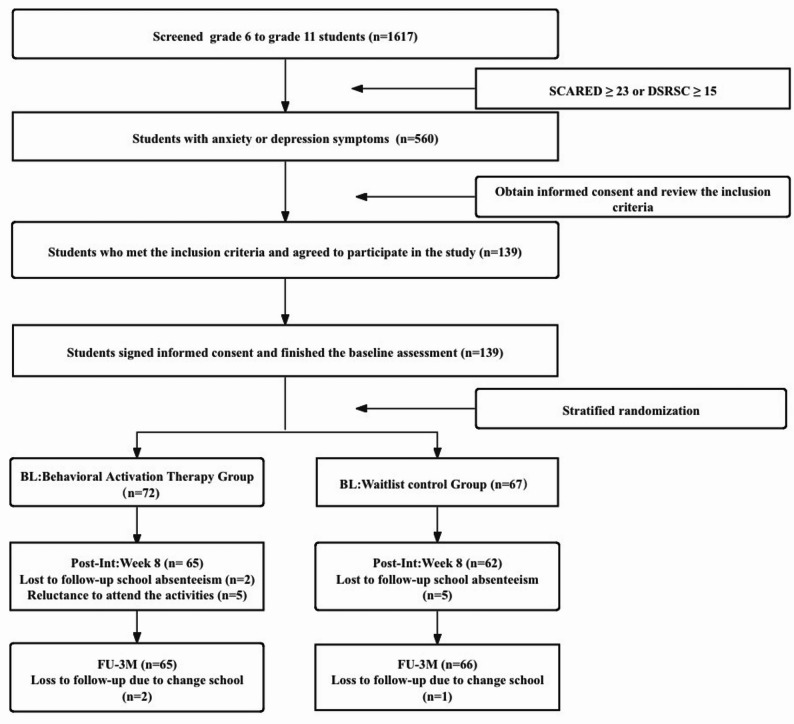
Study flowchart. *BL* baseline, *FU-3M* 3-month follow-up, *Post-Int* post-intervention

## Results

### Sample description

The participant flow is presented in Fig. 1. A total of 139 adolescents with clinically significant anxiety and/or depressive symptoms (*M*age = 14.38 years, *SD* = 1.50; 55.4% female) were randomized to either the GBAT group (*n* = 72) or the control group (*n* = 67). Baseline demographic and clinical characteristics are presented in Table 2. No significant between-group differences in depression severity, anxiety symptoms, gender distribution, parental marital status, or parental educational attainment were observed.

Five participants (6.90%) discontinued the intervention before Session 5, and 67 participants (93.06%) completed all eight sessions. Independent samples *t*-tests indicated no significant baseline differences between participants who completed the intervention versus those who discontinued early in SCARED (*t* (137) = 0.93, *p* = 0.35) or DSRSC (*t* (137) = − 1.21, *p* = 0.23). Post-intervention data were unavailable for two additional participants due to school absence during assessment. At the 3-month follow-up, two additional participants were lost to follow-up due to school transfers, resulting in complete data for 63 participants (87.5%) across all timepoints.

**Table 2:** Participant demographic and anxiety and depression characteristics baseline

	GBAT (*n* = 72)	WL (*n* = 67)	t/χ2	*P*
Age	14.45 ± 1.53	14.37 ± 1.50	0.31	0.76
SCARED	29.19 ± 10.51	29.58 ± 13.14	–0.20	0.85
DSRSC	13.48 ± 5.35	13.16 ± 4.81	0.37	0.71
SCARED ≥ 23	58	52	0.17	0.74
SCARED < 12	1	5	3.10	0.11
DSRSC ≥ 15	34	34	0.18	0.68
Gender			0.41	0.61
Male	34 (47.22%)	28 (41.79%)		
Female	38 (52.78%)	39 (58.21%)		
Grade			0.35	0.99
Grade 6	13	13		
Grade 7	13	14		
Grade 8	15	14		
Grade 10	15	12		
Grade 11	16	14		
Parental Marital Status			0.46	0.80
Married	63	56		
Divorced	8	10		
One parent deceased	1	1		
Maternal Educational Attainment			1.23	0.87
Middle school or below	4	2		
High school or equivalent	17	19		
Associate degree	16	12		
Bachelor’s degree	26	25		
Master’s degree or higher	9	9		
Paternal Educational Attainment			3.15	0.53
Middle school or below	3	3		
High school or equivalent	18	13		
Associate degree	12	18		
Bachelor’s degree	29	25		
Master’s degree or higher	10	8		

*DSRSC* Depression Self-Rating Scale for Children, *GBAT* Group Behavioral Activation Therapy, *SCARED* Screen for Child Anxiety Related Emotional Disorders, *WL* waitlist control

### Primary and anxiety symptom outcomes

An independent samples *t*-test revealed a significant reduction in SCARED scores following the GBAT intervention, *t*(135) = 2.44, *MD* = 4.20, *95% CI* [0.79, 7.60], *p* = 0.016, *Cohen’s d = 0.42.*

**Fig. 2:**
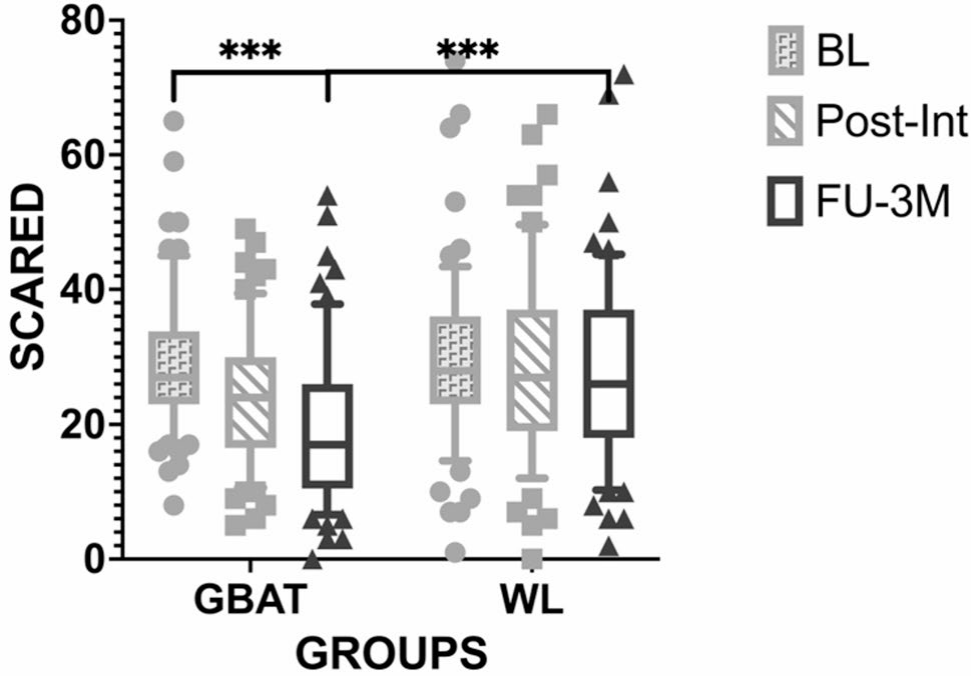
Changes in anxiety symptom scores measured by SCARED across study timepoints by group. Anxiety symptom scores were measured using the SCARED in the GBAT (*n* = 72) and WL (*n* = 67) groups at baseline, post-intervention, and 3-month follow-up. Data are presented as mean ± 90% confidence intervals (error bars). ***p < 0.001 for the comparison between baseline and 3-month follow-up within the GBAT group. *BL* baseline, *FU-3 M* 3-month follow-up, *GBAT* group-based behavioral activation therapy, *Post-Int* post-intervention, *SCARED* Screen for Child Anxiety Related Emotional Disorders, *WL* waitlist control

Linear mixed-effects modeling identified statistically significant main effects for both time and group allocation on SCARED scores (Table 3). Post hoc analyses indicated that symptoms improved significantly at the 3-month follow-up (follow-up vs. baseline: *MD* = 8.69, *95% CI* [3.58, 13.80], *p* < 0.001, *Cohen’s d* = 0.82; Fig. 2). Critically, participants in the GBAT group exhibited significantly lower anxiety symptoms than waitlist controls at follow-up (between-group *MD* = 7.33, *95% CI* [3.06, 11.60], *p* < 0.001).

Responder analysis (defined as ≥ 50% symptom reduction) demonstrated superior outcomes for GBAT relative to waitlist controls at 3 months, with significantly higher response and remission rates (Table 4).

### Depressive symptom outcomes

**Fig. 3:**
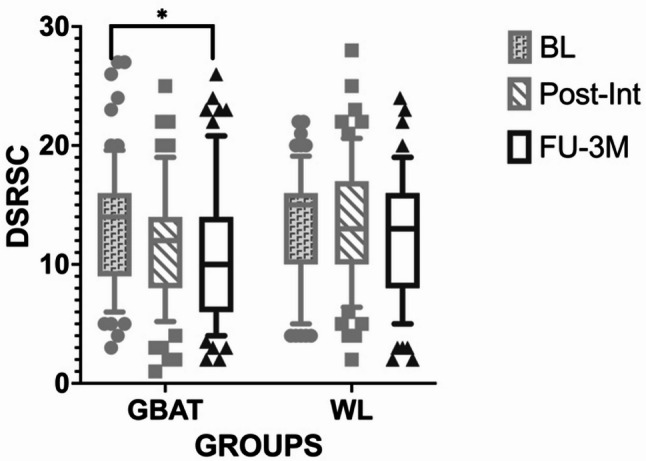
Changes in depressive symptom scores measured by DSRSC across study timepoints by group. Depressive symptom scores were measured using DSRSC in the GBAT (*n* = 72) and WL (*n* = 67) groups at baseline, post-intervention, and 3-month follow-up. Data are presented as mean ± 90% confidence intervals (error bars). * *p* < 0.005 for the comparison between baseline and 3-month follow-up within the GBAT group. *BL* baseline, *DSRSC* Depression Self-Rating Scale for Children, *FU-3 M* 3-month follow-up, *GBAT* group-based behavioral activation therapy, *Post-Int* post-intervention, *WL* waitlist control

Linear mixed-effects modeling similarly identified statistically significant main effects of both time and group on DSRSC scores (Table 3). Longitudinal analysis indicated a statistically significant reduction in depressive symptoms within the GBAT group at the 3-month follow-up relative to baseline (*MD* = 2.28, *95% CI* [0.10, 4.46], *p* = 0.037, *Cohen’s d* = 0.43; Fig. 3). However, between-group comparisons failed to achieve statistical significance at either the post-intervention (*MD* = 1.34, *p* = 0.156) or 3-month follow-up assessments (*MD* = 1.48, *p* = 0.111).

**Table 3:** Linear mixed-effects models for SCARED and DSRSC scores

Measure	Timepoint	Group		Outcome		
		GBAT (*n* = 72) Mean ± SD	WL (*n* = 67) Mean ± SD	Time Effect F(df) *p*	Group Effect F(df) *p*	Time*Group effect F(df) *p*
SCARED	BL	29.19 ± 10.51	29.58 ± 13.14	*F*(1,397) = 7.64 *p* < 0.001	*F*(1,397) = 10. 60 *p* = 0.001	*F*(2,397) = 1.84 *p* = 0.161
	Post-Int	24.38 ± 10.26	27.54 ± 14.34			
	FU-3 M	19.89 ± 12.01	27.22 ± 14.53			
DSRSC	BL	13.48 ± 5.35	13.16 ± 4.81	*F*(1,397) = 3.77 *p* = 0.024	*F*(1,397) = 4.235 *p* = 0.040	F(2,397) = 0.36 *p* = 0.697
	Post-Int	11.88 ± 4.9	13.21 ± 5.09			
	FU-3 M	10.79 ± 5.67	12.27 ± 5.29			

*BL* baseline, *DSRSC* Depression Self-Rating Scale for Children, *FU-3M* 3-month follow-up, *GBAT* group-based behavioral activation therapy, *Post-Int* post-intervention, *SCARED* Screen for Child Anxiety Related Emotional Disorders, *SD* standard deviation, *WL* waitlist control

**Table 4:** Between-group differences in response and remission rates

Timepoint	Outcome	Group		χ2	*p*
		GBAT *n* (%)	WL *n* (%)		
Post-intervention	Response			0.83	0.449
	SCARED reduce rate ≥ 50%	11 (16.92)	7 (11.29)		
	SCARED reduce rate < 50%	54 (83.08)	55 (88.71)		
	Remission			0.46	0.605
	SCARED total score ≤ 12	10 (15.38)	7 (11.29)		
	SCARED total score > 12	55 (84.62)	55 (88.71)		
3-month follow-up	Response			8.39	0.004
	SCARED reduce rate ≥ 50%	23 (35.38)	9 (13.64)		
	SCARED reduce rate < 50%	42 (64.62)	57 (86.36)		
	Remission			5.60	0.021
	SCARED total score ≤ 12	25 (38.46)	13 (19.70)		
	SCARED total score > 12	40 (61.54)	53 (80.30)		

“Response” is defined as a ≥ 50% reduction in SCARED total score from baseline; “Remission” is defined as a SCARED total score ≤ 12. All comparisons were conducted using χ^2^ tests with α = 0.05. *GBAT* Group Behavioral Activation Therapy, *SCARED* Screen for Child Anxiety Related Emotional Disorders, *WL* Waitlist control

Per-protocol analysis revealed significant main effects of time (*F* (2) = 15.35, *p* < 0.001, *η²* = 0.11), group (*F* (1) = 5.58, *p* = 0.02, *η²* = 0.04), and group×time interaction (*F* (2) = 3.67, *p* = 0.027, *η²* = 0.029) on SCARED scores (Table 5). Post hoc comparisons identified progressive symptom reductions within the GBAT cohort: from baseline to post-intervention (*MD* = 4.03, *95% CI* [1.22, 6.84], *p* = 0.005, *Cohen’s d* = 0.42), with further significant reductions observed at 3-month follow-up compared to both baseline (*MD* = 8.55, *95% CI* [5.28, 11.83], *p* < 0.001, *Cohen’s d* = 0.83) and post-intervention (*MD* = 4.53, *95% CI* [1.76, 7.29], *p* = 0.02, *Cohen’s d* = 0.41). Between-group analyses revealed significantly lower symptomatology in GBAT participants compared to controls exclusively at 3-month follow-up (*MD* = 7.69, *95% CI* [2.92, 12.46], *p* = 0.002, *Cohen’s d* = 0.57). For DSRSC, a significant main effect of time (*F* (2) = 7.88, *p* < 0.001, *η²* = 0.06) was observed, corresponding to modest improvement at 3-month follow-up compared to baseline (*MD* = 1.80, *95% CI* [0.76, 2.84], *p* < 0.001, *Cohen’s d* = 0.31), with no significant group or interaction effects (all *p* > 0.05). The analyses of both the intention-to-treat and per-protocol datasets demonstrated consistent trends.

**Table 5:** Repeated-measures ANOVA results for anxiety (SCARED) and depression (DSRSC) scores

Measure	Timepoint	Group		Outcome		
		GBAT (*n* = 63)	WL (*n* = 61)	Time effect F(df), *p*, *η²*	Group effect F(df), *p*,* η²*	Time*Group effect F(df), *p*,* η²*
SCARED	BL	28.29 ± 8.62	30.59 ± 13.34	*F(*2) = 15.346 *p* < 0.001 *η²* = 0.11	*F(*1) = 5.578 *p* = 0.02 *η²* = 0.044	*F*(2) = 3.674 *p* = 0.027 *η²* = 0.029
	Post-Int	24.27 ± 10.41	27.47 ± 14.45			
	FU-3 M	19.74 ± 11.81	27.43 ± 14.89			
DSRSC	BL	12.57 ± 5.50	13.75 ± 4.59	*F*(2) = 7.878 *p* < 0.001 *η²* = 0.061	*F*(1) = 2.684 *p* = 0.104 *η²* = 0.022	*F*(2) = 0.14 *p* = 0.869 *η²* = 0.001
	Post-Int	11.84 ± 5.24	13.20 ± 5.34			
	FU-3 M	10.77 ± 5.99	12.38 ± 5.43			

*BL* baseline, *DSRSC* Depression Self-Rating Scale for Children, *FU-3M* 3-month follow-up, *GBAT* group-based behavioral activation therapy, *Post-Int* post-intervention, *SCARED* Screen for Child Anxiety Related Emotional Disorders, *WL* Waitlist control, *η*^*2*^ partial eta squared

## Discussion

This quasi-experimental trial demonstrates that GBAT, when delivered by school mental health personnel, produced statistically significant and clinically meaningful reductions in anxiety and depressive symptoms among Chinese adolescents. Longitudinal assessment revealed that the 8-week intervention yielded progressive improvements in both anxiety and depressive symptoms, with therapeutic benefits maintained at the 3-month follow-up. Notably, anxiety symptom reduction demonstrated a large effect size (*Cohen’s d* = 0.82), while improvements in depressive symptoms exhibited a medium effect (*Cohen’s d* = 0.43). This study achieved a completion rate of 93.06% in the GBAT group, slightly superior to that reported in other school-based group cognitive behavioral therapy programs [40]. Collectively, these findings support the feasibility and effectiveness of GBAT delivered by trained school mental health personnel in educational settings, representing the first empirical validation of this implementation model in China’s adolescent mental health context.

Within the context of escalating mental health challenges among Chinese adolescents, compounded by severe service shortages [4, 41], these findings strengthen the empirical foundation supporting the broader implementation of school-based preventive interventions. Compared with traditional psychiatrist- or psychologist-delivered individual therapy, the GBAT paradigm offers potential cost-effectiveness and substantially improved accessibility. School mental health personnel embedded within educational systems offer distinct implementation advantages, including enhanced capacity for early detection of subclinical symptoms, increased student engagement due to reduced stigma compared with specialized mental health services [22, 40], and operational alignment with China’s educational infrastructure. This model enables the systematic integration of mental health support into academic routines, thereby directly addressing international priorities for early identification and early intervention [42, 43].

This study contributes to the growing empirical evidence supporting the efficacy of BA in mitigating adolescent anxiety and depression through targeting avoidance behavior patterns, consistent with established literature [44, 45]. The observed reduction in depressive symptoms was less pronounced than that reported by Andersson et al. [46], possibly due to differences in parental involvement across study designs. A robust body of clinical evidence demonstrates that parent-involved strategies significantly enhance treatment outcomes for children and adolescents [47–49]. Mechanistically, parental involvement may facilitate improvement by modifying maladaptive family environments (e.g., reducing family conflict and over-accommodation) and by optimizing parenting behaviors (e.g., increasing warmth and acceptance while decreasing criticism and blame) [49]. These changes enable parents to better support their child’s skill practice in daily life, promoting generalization and consolidation of these skills across contexts [50]. However, for parental involvement to be effective, it must extend beyond basic psychoeducation and precisely target the core maintaining factors of specific psychological issues [50]. Its implementation is often challenged by practical barriers such as time constraints, stigma, and parental attribution of responsibility [16]. Conversely, our intervention yielded a large effect size for anxiety symptoms (*Cohen’s d* = 0.82). This may be attributable to the substantial therapeutic effect of the intervention on anxiety symptoms, which achieved clinically significant improvement even without parental involvement.

Although prior research on the efficacy of BA for adolescent anxiety remains limited, our findings demonstrate significantly greater anxiety reduction relative to depressive symptoms, corroborating Chu et al.’s observations [34]. This differential treatment response may stem from the intervention’s underlying mechanistic architecture. The core components—systematic targeting of avoidance patterns followed by problem-solving and exposure techniques—directly engage the fundamental pathology of anxiety. Exposure therapy facilitates graded confrontation with anxiety-provoking stimuli, effectively reducing threat hypervigilance while enhancing self-efficacy and fear regulation capacities [51, 52]. These mechanisms address central pathways in anxiety pathogenesis and likely account for the superior improvements observed. In contrast, the core psychopathological features of depression—including anhedonia, motivational deficits, and maladaptive cognitive schemas—are less directly targeted by exposure-based techniques [53], although exposure can still contribute to improvements in depressive symptoms [54]. Furthermore, while problem-solving training enhances stress coping capacity, particularly in reducing anxiety-related helplessness, it is less effective for depression’s more entrenched emotional-cognitive patterns [55]. Thus, the greater reduction in anxiety symptoms likely reflects the intervention’s direct engagement of disorder-specific mechanisms. Methodological factors may also account for the greater anxiety improvements observed in this study compared with Chu et al. [34], despite the use of a comparable BA protocol. In particular, baseline differences in symptom severity may have contributed, as higher initial symptom levels are often associated with an attenuated treatment response [55].

Therapeutic effects were significantly greater at the 3-month follow-up compared to the immediate post-intervention assessment. This delayed improvement may reflect the theoretically informed sequencing of intervention components: initial modules emphasizing life goal assessment, symptom psychoeducation, and behavioral functional analysis demonstrated limited efficacy in symptom reduction, whereas later modules incorporating problem-solving therapy and exposure therapy yielded substantially greater clinical improvements [56, 57]. Exposure therapy requires repeated practice with progressively challenging tasks to achieve enduring effects [58]. Moreover, problem-solving therapy may enhance participants’ experience and confidence in problem-solving through changes in solution approaches or problem orientation, thereby increasing hopefulness about managing future challenges and reducing avoidance behaviors [59]. These changes occur gradually and therefore require time to consolidate. To maximize the effectiveness of these interventions, therapists must allocate sufficient treatment time and sessions for participants to systematically practice exposure and problem-solving techniques while providing timely feedback to positively reinforce their adaptive coping strategies.

The design and implementation of this study were carefully adapted to the sociocultural context of China. The intervention was delivered within the framework of the Chinese educational system by school mental health personnel who hold formal teaching positions. In China, such personnel typically assume multiple roles—including academic instruction, student counseling, and psychological intervention—and some also undertake administrative duties [60]. This institutional arrangement differs from the multi-tiered systems of support commonly implemented in school-based interventions in Western developed countries [61]. Given that the multifaceted role of school mental health personnel might discourage students’ self-disclosure, a cross-school intervention approach was adopted to mitigate this potential influence on intervention effectiveness. Furthermore, the program was integrated into the regular school curriculum as an extracurricular club activity. To reduce students’ potential reservations associated with traditional classroom dynamics, such as lecture-style instruction and evaluative atmospheres, the standard “podium-seating” arrangement was replaced with a round-table group format. This setup aimed to foster a more egalitarian and open interactive environment. In addition to these structural adaptations, group facilitators received comprehensive training and ongoing supervision. Training emphasized the ability to promptly recognize participants’ engagement levels and intervene accordingly—for instance, by normalizing negative emotions or proactively introducing common distressing scenarios experienced by students. These strategies were intended to create a safe and supportive environment for self-expression. Despite the curriculum-embedded approach aimed at improving acceptability, the final participation rate was only 24.82%, likely influenced by mental health stigma and the predominant academic-oriented mindset.

### Limitations

This study has several limitations, which we have categorized into methodological and contextual/logistical domains to inform future research. Methodologically, although the intervention was delivered in accordance with the treatment manual, the absence of objective monitoring (e.g., audio/video recordings) limits our ability to assess adherence and potential variability in implementation. In addition, the intervention and control groups were situated within the same schools, which raises the possibility of contamination and may have attenuated the observed effects. Reliance solely on student self-reports for primary outcomes also raises the possibility of response biases. Contextually and logistically, the sample was drawn from a single administrative district in Shanghai, limiting the geographical and socioeconomic generalizability of the findings.

## Conclusion and suggestion

These findings provide preliminary but encouraging evidence for the effectiveness and feasibility of GBAT delivered by school mental health personnel in alleviating anxiety and depressive symptoms among Chinese adolescents. By demonstrating successful implementation within a real-world educational setting, this study lays a crucial groundwork for the broader application of GBAT as a scalable early-intervention model in schools. To advance these promising findings, future research should prioritize large-scale trials that recruit more diverse and geographically representative samples. When coupled with multi-informant assessment protocols, such studies will be well positioned to confirm effectiveness, elucidate the underlying mechanisms of change, and validate the long-term sustained impact of this parsimonious intervention model.

## Data Availability

No datasets were generated or analysed during the current study.
